# Analysis of an Intelligence Dataset

**DOI:** 10.3390/jintelligence8040039

**Published:** 2020-11-19

**Authors:** Nils Myszkowski

**Affiliations:** Department of Psychology, Pace University, New York, NY 10038, USA; nmyszkowski@pace.edu

It is perhaps popular belief—at least among non-psychometricians—that there is a unique or standard way to investigate the psychometric qualities of tests. If anything, the present Special Issue demonstrates that it is not the case. On the contrary, this Special Issue on the “analysis of an intelligence dataset” is, in my opinion, a window to the present vividness of the field of psychometrics.

Much like an invitation to revisit a story with various styles or with various points of view, this Special Issue was opened to contributions that offered extensions or reanalyses of a single—and somewhat simple—dataset, which had been recently published. The dataset was from a recent paper (Myszkowski and Storme 2018), and contained responses from 499 adults to a non-verbal logical reasoning multiple-choice test, the SPM–LS, which consists of the Last Series of Raven’s Standard Progressive Matrices (Raven 1941). The SPM–LS is further discussed in the original paper (as well as through the investigations presented in this Special Issue), and most researchers in the field are likely familiar with the Standard Progressive Matrices. The SPM–LS is simply a proposition to use the last series of the test as a standalone test. A minimal description of the SPM–LS would probably characterize it as a theoretically unidimensional measure—in the sense that one ability is tentatively measured—comprised of 12 pass-fail non-verbal items of (tentatively) increasing difficulty. Here, I refer to the pass-fail responses as the binary responses, and the full responses (including which distractor was selected) as the polytomous responses. In the original paper, a number of analyses had been used, including exploratory factor analysis with parallel analysis, confirmatory factor analyses using a structural equation modeling framework, binary logistic item response theory models (1-, 2-, 3- and 4- parameter models), and polytomous (unordered) item response theory models, including the nominal response model (Bock 1972) and nested logit models (Suh and Bolt 2010). In spite of how extensive the original analysis may have seemed, the contributions of this Special Issue present several extensions to our analyses.

I will now briefly introduce the different contributions of the Special Issue, in chronoligical order of publication. In their paper, Garcia-Garzon et al. (2019) propose an extensive reanalysis of the dimensionality of the SPM–LS, using a large variety of techniques, including bifactor models and exploratory graph analysis. Storme et al. (2019) later find that the reliability boosting strategy proposed in the original paper—which consisted of using nested logit models (Suh and Bolt 2010) to recover information from distractor information—is useful in other contexts, by using the example on a logical reasoning test applied in a personnel selection context. Moreover, Bürkner (2020) later presents how to use his R Bayesian multilevel modeling package brms (Bürkner 2017) in order to estimate various binary item response theory models, and compares the results with the frequentist approach used in the original paper with the item response theory package mirt (Chalmers 2012). Furthermore, Forthmann et al. (2020) later proposed a new procedure that can be used to detect (or select) items that could present discriminating distractors (i.e., items for which distractor responses could be used to extract additional information). In addition, Partchev (2020) then discusses issues that relate to the use of distractor information to extract information on ability in multiple choice tests, in particular in the context of cognitive assessment, and presents how to use the R package dexter (Maris et al. 2020) to study the binary responses and distractors of the SPM–LS. I then present an analysis of the SPM–LS (especially of its monotonicity) using (mostly) the framework of Mokken scale analysis (Mokken 1971). Finally, Robitzsch (2020) proposes new procedures for latent class analysis applied on the polytomous responses, combined with regularization to obtain models of parsimonious complexity.

It is interesting to note that, in spite of the relative straightforwardness of the task and the relative simplicity of the dataset—which in the end, contains answers to a few pass-fail items in a (theoretically) unidimensional instrument—the contributions of this Special Issue offer a lot of original and new perspectives on analyzing intelligence test data. Admittedly, much like the story retold 99 times in Queneau’s *Exercices de style*, the dataset reanalysed in this Special Issue is, in of itself, of moderate interest. Nevertheless, the variety, breadth and complementarity of the procedures used, proposed and described here clearly demonstrate the creative nature of the field, giving an echo to the proposition by Thissen (2001) to see artistic value in psychometric engineering. I would like to thank Paul De Boeck for proposing the topic of this Special Issue and inviting me to act as guest editor, as well as the authors and reviewers of the articles published in this issue for their excellent contributions. I hope that the readers of *Journal of Intelligence* will find as much interest in them as I do.
